# An old friend with a new face: tRNA-derived small RNAs with big regulatory potential in cancer biology

**DOI:** 10.1038/s41416-023-02191-4

**Published:** 2023-02-09

**Authors:** Arianna Di Fazio, Monika Gullerova

**Affiliations:** Sir William Dunn School of Pathology, University of Oxford, Oxford, OX1 3RE UK

**Keywords:** Small RNAs, Cancer epigenetics

## Abstract

Transfer RNAs (tRNAs) are small non-coding RNAs (sncRNAs) essential for protein translation. Emerging evidence suggests that tRNAs can also be processed into smaller fragments, tRNA-derived small RNAs (tsRNAs), a novel class of sncRNAs with powerful applications and high biological relevance to cancer. tsRNAs biogenesis is heterogeneous and involves different ribonucleases, such as Angiogenin and Dicer. For many years, tsRNAs were thought to be just degradation products. However, accumulating evidence shows their roles in gene expression: either directly via destabilising the mRNA or the ribosomal machinery, or indirectly via regulating the expression of ribosomal components. Furthermore, tsRNAs participate in various biological processes linked to cancer, including apoptosis, cell cycle, immune response, and retroviral insertion into the human genome. It is emerging that tsRNAs have significant therapeutic potential. Endogenous tsRNAs can be used as cancer biomarkers, while synthetic tsRNAs and antisense oligonucleotides can be employed to regulate gene expression. In this review, we are recapitulating the regulatory roles of tsRNAs, with a focus on cancer biology.

## Introduction

In the central dogma of molecular biology, genetic information is translated from DNA to proteins by means of messenger RNA (mRNAs). Transfer RNA (tRNA) is a universal adaptor molecule and decoder of genetic information: tRNAs translate mRNAs into proteins by carrying amino acids to the growing polypeptide chain at the ribosomal complex. Discovered more than 50 years ago, tRNAs have been chemically, structurally, and functionally well-characterised [1, 2]. Despite our extensive knowledge of tRNA biology, it is only recently emerging that tRNAs can be fragmented into functional small non-coding RNAs (sncRNAs), named tRNA-derived small RNAs (tsRNAs).

tsRNAs were first identified in the urine samples of cancer patients in the 1970s and originally thought to be clearance by-products [3, 4]. Advanced next-generation sequencing (NGS) techniques, with increased depth and means to overcome the technical constraints dictated by RNA chemical modifications [5, 6], allowed identification of tsRNAs as a bona fide class of sncRNAs, deliberately generated by the specific action of ribonucleases [7]. Highly conserved tsRNAs have been identified in silico and in vivo within various species, including human, mouse and zebrafish [7–11]. Recently, the identification of tsRNA sequences and the characterisation of their relevance to cancer and other pathologies has increased [12–14].

The sequence diversity of tsRNAs arises from the complexity of the tRNAomes. Despite the limited number of DNA codons, there are thousands of tRNA genes across eukaryotic organisms [15, 16]. Humans have around 500 genes encoding tRNAs, about half of which are inactive pseudogenes [17]. This gene redundancy could be explained by the existence of tRNA isoacceptors (tRNAs charged with the same amino acid but with different anticodons) and isodecoders (tRNAs with the same anticodon but with different body structures) [18]. Interestingly, somatic mutations in some of the tRNA genes have been documented to cause various diseases, suggesting that each tRNA may play a non-canonical, sequence-dependent role (for example, as the source of tsRNAs) that cannot be compensated by other tRNAs [19]. Alternatively, tRNA isodecoders may be expressed in a tissue-specific manner [18, 20].

Cancer is one of the most lethal and challenging pathologies, causing millions of deaths every year. Dysregulation of tsRNA expression levels has been associated with many cancer pathologies [21]. sncRNAs, including micro-RNA (miRNAs) and Piwi-interacting RNA (piRNAs) are known to participate in cancer onset and progression [22, 23]. tsRNAs share many structural and functional characteristics with miRNAs and piRNAs. Hence, it is not surprising that there is increasing evidence for tsRNA regulatory roles in many cellular contexts linked to cancer. Therefore, it is fundamental to understand the regulatory roles of tsRNAs and how these can be used for therapeutic and diagnostic cancer applications.

In this review, we report the current classification of tsRNA subclasses and their biogenesis. We also summarise known mechanisms of tsRNAs dysregulation and tsRNA-driven mechanisms of cancer onset and progression. Finally, we discuss examples of tsRNAs involved in cancer and conclude with an overview of tsRNA-based therapeutic applications.

### tsRNAs classification and biogenesis

A universal nomenclature system for tRNA-derived small RNA does not yet exist. This class of sncRNAs is referred to as ‘tsRNAs’ but also, tRNA fragments ‘tRFs’or tRNA-derived RNA ‘tdRNAs’. The name ‘tsRNA’ is becoming increasingly popular in the field. Hence, we have used ‘tsRNA’ in this review.

**Fig. 1:**
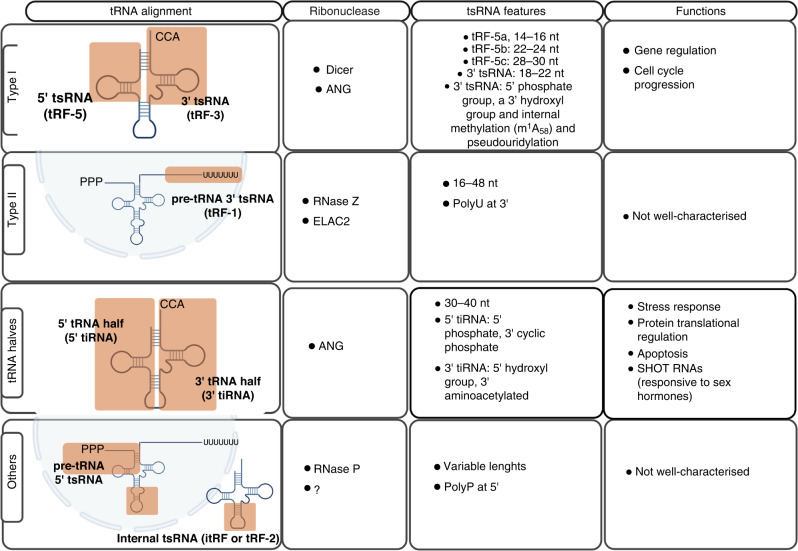
Summary of tsRNAs classification and features. First column: tsRNAs are classified according to alignment to tRNA precursors. Type I tsRNAs are generated by cleavage of 5’ or 3’ mature tRNA ends; type II tsRNAs align with 3’ trailer sequence of pre-tRNAs. tRNA halves align to half of the mature tRNA molecules. Other tsRNA types are generated from 5’ leader sequence of pre-tRNAs or internal tRNA or pre-tRNA sequences. The second column summarises known ribonucleases involved in tsRNA biogenesis. Features of each tsRNA type including chemical modifications and length are highlighted in the third column. Finally, some general functions of tsRNA types related to cancer biology are reported in last column. Image was created with Biorender.com. tsRNA tRNA-derived small RNA, ANG Angiogenin, m1A N1-methyladenosine, RNase Z ribonuclease Z, ELAC2 ElaC ribonuclease Z 2, SHOT RNA sex hormone-dependent tRNA-derived RNAs, RNase P ribonuclease P.

tsRNAs biogenesis can occur at various stages of tRNAs maturation. tRNA molecules are transcribed by RNA Polymerase III (Pol III) as pre-tRNAs [24]. During maturation, the pre-tRNA 5’ PPP- leader sequence is cleaved by RNAse P and the 3’ trailer-UUU sequence is cleaved by RNAse Z. Some tRNAs also undergo splicing of the intronic sequence, contained between the anticodon and variable length loop. Lastly, the addition of the -CCA triplet at the 3’ terminus marks tRNAs maturation [25]. Mature tRNAs are ~70–90 nucleotides (nt) long. tRNAs, as well as pre-tRNAs, are cleaved into tsRNAs via specific pathways. Depending on the tRNA precursor alignment and cleavage site, tsRNAs can be classified into several subclasses or types, as shown in Fig. 1.

Type I tsRNA are generated by cleavage of the mature tRNA at either the 5’ or 3’ end.

5’ tsRNAs (or tRFs-5) align to the 5′ end of tRNA and extend at least up to the D or the anticodon loop. 5’ tsRNAs are more abundant within specific ranges of nucleotide length. According to this, 5’ tsRNAs are further divided into tRF-5a, 14–16 nt (up to the D loop); tRF-5b, 22–24 nt long (up to the stem region between the D and anticodon loop); and tRF-5c, 28–30 nt long (up to the anticodon loop) [26]. However, 5’ tsRNAs with other lengths that do not fall into those subcategories have also been identified in prostate cancer cells and archaea, indicating that 5’ tsRNAs and their biogenesis may be more heterogenous than we first thought [27, 28].

3’ tsRNAs (or tRFs-3) originate from the 3′ end of mature tRNA, including the -CCA triplet and have a standardised length of 18–22 nt [26]. As mature tRNA molecules are decorated by several chemical modifications, 3’ tsRNAs can carry those chemical marks. The variety and roles of those chemical modifications on tsRNAs have been discussed before [29, 30]. 3’tsRNAs contain some consistent chemical modifications such as a 5’ phosphate group, a 3’ hydroxyl group, internal methylation (m^1^A_58_) and pseuodourydilation, that may have a role in 3’ tsRNA biogenesis.

Type II tsRNAs (or tRFs-1) match the 3’ end of the pre-tRNA trailer sequence and terminate with the polyU termination signal for Pol III [31]. Type II tsRNAs are generated by RNAse Z or ELAC2 and have heterogenous lengths between 16–48 nt [26, 31, 32].

Other tsRNAs that are not included in this classification are internal tRNA fragments (itRFs or tRFs-2), which align with the internal region around the anticodon loop of mature tRNAs and have variable lengths [33]. In addition, pre-tRNA 5’ leader sequence can also generate tsRNAs by RNAse P cleavage during tRNA maturation, which can include or exclude part of the 5’ mature tRNA up to the anticodon loop [34]. Lastly, tRNA-derived stress-induced small RNAs (tiRNAs), also known as tRNA halves, align with half of the mature tRNA molecule, are 30–40 nt long and can be divided into 5’ tiRNAs (with a 5’ phosphate and a 3’ cyclic phosphate) and 3’ tiRNAs (5’ hydroxylated and 3’ amino acetylated) [35–37].

At present, we lack a comprehensive understanding of tsRNAs biogenesis. To date, only a handful of ribonucleases have been reportedly able to cleave tRNA or pre-tRNA: RNase P [38], RNase Z [31], ELAC2 [32], Angiogenin (ANG)[37, 39, 40], Dicer [7, 41, 42] and in vitro RNase T2 [43]. It is likely that more tRNA nucleases and biogenesis pathways will be elucidated in the future. However, there is general agreement that tRNA halves are produced by cleavage in the anticodon of mature tRNAs by ANG, driven by the stress response or tRNA structural instability [37, 44]. ANG is also responsible for generating variable length, stress-induced 5’tsRNAs. Some tsRNAs and tRNA halves have been detected in ANG-depleted cells, suggesting that there may be alternative or compensatory pathways [40]. One study suggested that some tsRNAs are produced by Dicer, in the absence of ANG [45]. Interestingly, another study showed that RNAse T2 can also generate 5’ tsRNAs and tRNA halves in vitro [43].

There is controversy about Dicer being responsible for type I tsRNAs biogenesis. Some studies support tsRNAs Dicer dependency, especially of the 3’ tsRNAs [7, 9, 10, 31, 42, 46]. Whereas others endorse the idea that tsRNAs are Dicer independent, as the levels of tsRNAs remain unchanged in Dicer KO cells [47, 48]. Given that 3’ tsRNAs have miRNA-like features, such as length and chemical modifications at the 5’ and 3’ ends [31], they are likely to be generated by Dicer.

### Mechanisms of tsRNAs dysregulation

As discussed in the previous section, the biogenesis of tsRNAs is not fully elucidated, and even less is known about the regulation of tsRNA levels. Usually, tsRNAs have a constant expression level across healthy tissue samples but are dysregulated in many pathologies, including cancer [49, 50]. More than 400 tsRNA sequences have been annotated across NCI-60 and TCGA panels of cancer cell lines and collated into a database, tRFExplorer [51]. Databases, such as OncotRF and mintBase v 2.0, also offer an overview of tsRNAs dysregulation in various cancers [13, 14]. Various studies suggest a tsRNA tissue-specific cancer signature that could be valuable for biomarker and diagnostic applications [52, 53]. Increasing our knowledge on tsRNAs regulation is fundamental for developing therapeutic, diagnostic and prognostic strategies. Therefore, it is relevant to interrogate the causes of altered tsRNAs regulation.

#### Transcriptional and biogenesis-related dysregulation of tsRNAs

tRNA abundance is in some instances correlated with diseases [19]. In particular, cancer onset and progression are characterised by high metabolic and translation rates, hence large tRNA pools [54]. Zhang et al. identified a tsRNA (referred to as tRNA-Glu–derived piRNA or td-piR(Glu)), that is downregulated by interleukin-4 (IL-4) [55]. Since IL-4 is known to induce monocyte differentiation and, during this stage, Pol III transcriptional activity is strongly reduced, it is possible that td-piR(Glu) decreased levels may be a result of decreased level of tRNA-Glu precursor [55]. However, a study has shown that tsRNA abundances do not correlate with the availability of tRNA precursors alone but rather the activity of the amino acid that charges the tRNA with its amino acid [56]. This suggests that tsRNA biogenesis may be a regulated process.

Runt-related transcription factor 1 (RUNX1) is a transcription factor involved in the pathogenesis of cancer by regulating cellular processes, including apoptosis and cell proliferation. It is associated with overall poor patient prognosis [57]. Interestingly, a study has identified four tsRNAs that are responsive for the deregulation of RUNX1 [58]. It is not clear whether that could be caused directly on a transcriptional level, but it suggests that tsRNA levels could be altered by the activity of oncogenes.

It is also useful to investigate whether aberrant levels of tsRNA are the product of dysregulated ribonucleases that process tRNAs. Examination of cancer databases, such as The Human Protein Atlas, has shown ubiquitous expression of ELAC2/RNAse Z with no specificity for cancer cells. Therefore, the cellular availability of these ribonuclease has little effect on type II tsRNA levels [58]. Similarly, Dicer is generally poorly associated with cancer specificity, which suggests lack of oncogenic link between Dicer and tsRNA levels. ANG expression regulation is characterised by nuclear sequestration or cytoplasmic association with ribonuclease/angiogenin inhibitor 1 (RNH1). Upon RNH1 dissociation or release from the nucleus/nucleolus, ANG is activated [37]. It is possible that aberrant levels of tRNA halves could be caused by endogenous dysregulation of ANG, however, studies supporting this hypothesis are lacking at present.

#### Chemical modifications of tRNA

RNA is the most post-transcriptionally modified molecule, with over 150 different chemical modifications identified and extensively reviewed previously [59–62]. On average, each tRNA molecule contains 13 modifications [63]. Chemical modifications are catalysed by a growing set of proteins, called 'writers'. Each of them is responsible for modification of a few unique nucleotides. These chemical modifications are dynamic and reversible as they can be removed by 'erasers' and have signalling potential as they are recognised by 'readers' [64]. Chemical modifications on tRNAs play a role in maintaining the stability of RNA structures and processing of tRNAs into tsRNAs. Therefore, deregulation of writers, readers or erasers may be accountable for aberrant tsRNA levels. Among the many chemically diverse RNA modifications, queuosine is one example of those chemical marks been shown to protect tRNAs from cleavage [65]. Methylation [66, 67] and pseudouridylation [68] have been mostly implicated in the biogenesis of shorter fragments derived from tRNAs.

In humans, DNA methyltransferase 2 (DNMT2) and NOP2/Sun RNA methyltransferase family member 2 (NSUN2) are the prominent 5-methylcytosine (m^5^C) methyltransferases of ncRNAs [69]. Indeed, there is evidence for the protective role of NSUN2 and DNMT2-driven methylation against tRNA fragmentation [70]. DNMT2-driven hypomethylation has been previously associated with tRNA instability and the generation of tRNA fragments in stress response pathways by ANG [44]. Ablation of DNMT2 in mouse sperm has been associated with the increase of a set of tsRNAs [71, 72]. Similarly, tsRNAs biogenesis in Drosophila was responsive to DNMT2 activity [73]. NSUN2 has been reported to be upregulated in many cancers [74–79]. In addition, it has been suggested that NSUN2 is involved in vault RNA (vtRNA) processing into small vtRNAs, another class of sncRNAs with similar properties to tsRNAs [80, 81].

tRNA methyltransferase 2 homologue A and B (TRMT2A and TRMT2B) were identified as writers of 5-methyluridine (m^5^U_54_), which is another conserved tRNA chemical modification, on tRNAs. TRMT2A knockdown led to tRNAs hypomethylation and increased biogenesis of 5’ tRNA halves by ANG [82].

Finally, the reversion of m^5^C by the eraser Ten-Eleven-Translocation 2 (TET2) has been implicated in promoting tRNA translational activity and regulation of tsRNA biogenesis [83]. AlkB Homologue 3, (ALKBH3) is also a known RNA methylation eraser and it is upregulated in various cancers, including pancreatic, lung, colorectal and urothelial. ALKBH3 has been shown to demethylate tRNA 1-methyladenosine (m^1^A) and 3-methylcytidine (m^3^C) both in vitro and in vivo [84]. ALKBH3 demethylated tRNAs were more prone to processing by ANG [84].

Pseudouridylation is the isomerisation of the uracil nucleotide [59]. The Pseudouridine synthase (PUSs) family is responsible for catalysing this modification, especially PUS7 and PUS10 [68, 85]. Despite lacking clear evidence and mechanistic insight of PUS7/10 involvement, studies suggest that there is a connection between pseudouridylation, tsRNAs biogenesis and cancer progression [68, 86, 87].

#### Environmental stresses and intercellular signalling

Cells are exposed to many environmental insults that induce stress. One common stress response is a change in RNA metabolism and, consequently, reduced metabolic rates of protein synthesis [88]. As tRNAs are the mediator of protein translation, it is not surprising that a common stress response is the alteration of the cellular tRNA pools. tRNA 3’ end trimming is a known mechanism in *Trypanosoma brucei*, by which the organism makes tRNAs unable to perform the translation in response to stress [89]. In humans, tRNA fragmentation can be caused by oxidative stress [37]. ANG is the ribonuclease mostly associated with stress-induced tRNA halves biogenesis [90]. It has been proposed that exposure to heavy metals, such as arsenite, drives ANG, but also Dicer-mediated tRNA halves production [40, 45].

It should be kept in mind that despite being an effective way of reducing active tRNA pools among many organisms, tsRNA biogenesis should not be reduced to this function [91].

In addition to environmental stress factors, multicellular organisms rely on hormones and cytokines for intercellular communication. Deregulation of sex hormones is involved in breast and prostate cancer. A group of special tRNA halves is regulated by sex hormones, named sex hormones-dependent tRNA-derived RNA (SHOT RNA) [92]. Honda et al. showed that the addition of estradiol and dihydrotestosterone (DHT) to receptor-positive breast and prostate cancer cell lines (MCF-7, BT-474 and LNCa-FGC) resulted in increased levels of SHOT RNAs [92]. Interestingly, only 5’ SHOT RNAs were shown to promote cell proliferation [92].

### tsRNA-driven regulatory mechanisms in cancer

**Fig. 2:**
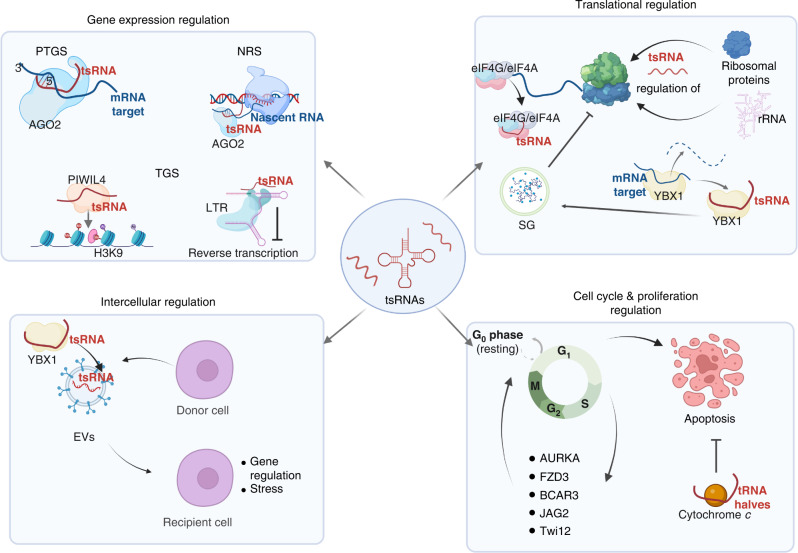
Summary of relevant tsRNA-driven mechanisms of gene expression regulation. tsRNAs mediate gene silencing. tsRNAs are loaded on Argonaute 2 (AGO2) and participate in post-transcriptional gene silencing (PTGS), and nascent RNA silencing (NRS) by targeting messenger (mRNA) and nascent RNA, respectively. tsRNAs also mediate transcriptional gene silencing (TGS) by recruitment of histone methyltransferases; and inhibition of long-terminal repeat (LTR) retrotranscription by competing with tRNA. tsRNAs mediate mechanisms of translational regulation by affecting ribosomal proteins and rRNA expression. In addition, they can stall protein translation by displacing the initiation factor eIF4G/eIF4A complex and inducing stress granules (SGs) formation; SGs formation are also induced via YBX1 binding. tsRNAs can displace YBX1 from mRNA transcripts and promote mRNA degradation. tsRNAs are involved in the regulation of the cell cycle and apoptosis by interfering with several signalling mechanisms. Lastly, tsRNAs are loaded in extracellular vesicles (EVs) and mediate mechanisms of regulation in recipient cells. Image was created with Biorender.com. PIWIL4 Piwi-Like RNA-mediated gene silencing 4, H3K9 histone H3 lysine 9, YBX1 Y box-binding protein, AURKA Aurora kinase A, FZD3 Frizzled Class Receptor 3, BCAR3 breast cancer anti-oestrogen resistance protein 3, JAG2 Jagged Canonical Notch Ligand 2, Twi12 tetrahymena Piwi protein.

tsRNAs roles in cancer biology have been linked to several biological mechanisms. In summary, tsRNAs can regulate gene expression at many levels by means of transcriptional and epigenetic regulation. They can additionally exercise translational regulation by affecting ribosomal machinery components and their functions. Moreover, tsRNAs participate in diverse cell signalling mechanisms to promote or inhibit cellular proliferation, as summarised in Fig. 2.

#### Gene expression regulation

Many cancers arise from aberrant oncogene regulation, which results in the overexpression of proto-oncogenes and downregulation of tumour suppressor genes. sncRNAs mediate gene silencing by RNA interference (RNAi) mechanisms. RNAi can be classified as post-transcriptional gene silencing (PTGS) [93, 94], or transcriptional gene silencing (TGS) [95, 96]. PTGS effectors assemble in the RNA-induced silencing complex (RISC). There are two ways of achieving gene silencing: via direct cleavage of mRNA by Argonaute 2 (AGO2) slicer activity, or by cytoplasmic translational repression [93, 94]. TGS, on the other hand, utilises the RNA-induced transcriptional silencing complex (RITSC) to recruit chromatin-modifying enzymes that induce chromatin condensation and repress transcription [95, 96]. Even though some studies have shown tsRNAs binding preference for AGO1, AGO3 and AGO4 [26, 31, 41], tsRNAs can also function in a miRNA-like fashion to guide cytoplasmic AGO2 in executing PTGS [41, 47, 97]. tsRNAs have more than once been mistakenly annotated as miRNAs, further supporting their roles in PTGS [9, 98]. Key examples of tsRNAs that drive PTGS of oncogenes are reported in Table 1.

**Table 1:** Examples of tsRNAs involved in cancer biology.

	Name	tRNA	tsRNA class	Mechanism	Reference
Proto-oncogenic	ts-53 (ts-3676)	Thr^AGT^	pre-tRNA 3’ tsRNA	Targets 3’ UTR of oncogene *TCL1* via AGO2	[101]
	tRF-3017A	Val^TAC^	3’ tsRNA	Silences tumour suppressor gene *NELL2* via RISC activity with AGO proteins	[150]
	3’tsRNA-Leu^CAG^	Leu^CAG^	3’ tsRNA	Unwinds mRNA of ribosomal proteins and promotes ribosomal biogenesis	[113]
	tRF-Leu^CAG^	Leu^CAG^	5’ tsRNA	Promotes cell cycle progression by interacting with AURKA	[117]
	tRF-1001	Ser^TGA^	pre-tRNA 3’ tsRNA	Depletion of tRF-1001 impairs cell proliferation and causes accumulation of cells in G2	[32]
Tumour suppressor	tRF-Glu-TTC-027	Glu^TTC^	not classified	Regulates MAPK signalling in gastric cancer cells	[151]
	tRF-24-V29K9UV3IU	Gln^TTG^	5’ tsRNA	Silences GPR78 by destabilising the 3’ UTR of its mRNA via AGO2; inhibits tumour progression	[97]
	tRF-3 CU1276	Gly^GCC^	3’ tsRNA	Regulates RPA1, master regulator or DNA damage response; drives arrest of Burkitt lymphoma cell proliferation	[41]
	tRF-5 Glu	Glu^CTC^	5’ tsRNA	Downregulates BCAR3; suppresses ovarian cancer cell proliferation	[122]
	tRF/miR1280	Leu	3’ tsRNA	Interacts with JAG2 and downregulates Notch signalling pathway	[121]
	AS-tDR-001430	Val^CAC^	5’ tRNA half	Binds FZD3, suppresses the Wnt/*β*-catenin signalling pathway and inhibits cell proliferation	[120]

Furthermore, we have recently proposed a novel mechanism of nuclear nascent RNA silencing (NRS) [42]. In this mechanism, Dicer-dependent tsRNAs guide AGO2 to specific target sites located in intronic regions of the nascent RNA, resulting in degradation of nascent transcripts and gene silencing [42]. We have identified ~1000 genes that are predicted to be regulated by NRS, including many proto-oncogenes such as *Bcl2* and *Egfr* [42].

Therefore, tsRNAs could be considered a new class of siRNA, driving various forms of gene silencing. It would be interesting to investigate whether, similarly to miRNAs, tsRNAs could also drive gene activation, perhaps targeting promoters of protein-coding genes.

#### Epigenetic and structural genome regulation

Epigenetic mechanisms, in particular aberrant methylation marks in DNA promoter regions, have been linked to disturbed oncogene homoeostasis and the onset of cancer [99, 100]. A few pioneer studies have identified roles for tsRNA in epigenetic regulation mechanisms, either in a Piwi-dependent or independent manner [55, 99], proposing potential TGS roles for tsRNAs.

tsRNAs can be bound to the PIWI proteins, which are well-known epigenetic players in cancer cells. For example, type II tsRNA molecules ts-4521 and ts-3676, (now known as ts-101 and ts-53, respectively) were identified to be bound to PIWIL2 [101]. Zhang et al. showed an epigenetic mechanism in human monocytes. Here, a tsRNA with PIWI binding properties binds to the PIWIL4. This drives the recruitment of H3K9 methyltransferases, SUV39H1 and SETDB1, and heterochromatin protein 1*β* (HP1*β*) to the promoter region of *CD1A*, causing inhibition of its transcription [55].

In addition, tsRNAs can regulate transposable elements and chromatin accessibility in a PIWI-independent manner [99]. Events of retroviral transcription and transposon insertion can lead to random integration in the genome, resulting in insertional mutations and potentially cancer [102]. 3’ tsRNAs-CCA were found to inhibit long-terminal repeat (LTR)-retrotransposon reverse transcription by competing with full-length tRNA, which can act as a primer for viral reverse transcription [103].

Regulation of the structural organisation of chromatin is a well-known strategy for gene silencing. Chromatin modifiers can deposit modifications on histones, leading to chromatin condensation and the formation of heterochromatin, which is inaccessible to transcriptional effectors. Conversely, chromatin relaxation to euchromatin promotes transcription factor accessibility and hence gene expression [104]. A recent study identified a 5’ tRNA half from tRNA-Gly^GCC^ that is involved in the production of Cajal bodies. These contain a variety of sncRNAs, including U7 snRNAs [105]. U7 snRNAs affect histone expression by binding histone downstream element, therefore altering chromatin status [105].

Altogether, tsRNAs can drive histone modifications, leading to chromatin remodelling. Furthermore, their inhibitory role in retrotransposon transcription could be relevant to gene amplification, one of the drivers of cancer.

#### Translational regulation

Metabolic reprogramming is a hallmark of cancer. Cancer cells have a high energetic demand due to enhanced protein production, required to support dysregulated cell growth and division [106]. In addition to transcriptional and post-transcriptional regulation, tsRNAs can regulate protein expression at the translational level, via multiple mechanisms. Interestingly, many tsRNAs with translational inhibitory functions were reported to have a GG-conserved motif at nucleotide 17–18 or 18–19 [107]. This suggests tRNA-protein binding or structural roles for these tsRNAs.

In fact, many mechanisms of translational regulation involve tsRNAs binding to and sequestering protein components of the translational machinery, as well as the formation of non-canonical RNA–protein complexes. For instance, tRNA halves can inhibit translation initiation by interacting with the cap binding eIF4F complex and also displacing the eIF4G/eIF4A complex from capped and uncapped mRNAs. In addition, this mechanism induces stress granule (SG) formation and, consequently, stalled protein translation [90].

tsRNAs can influence translational rates by binding Y box-binding protein (YBX1), a multifunctional RNA/DNA binding protein [108]. It was also reported that in breast cancer cells, tsRNAs can induce tumour suppressor effects via displacement of YBX1 from the mRNAs of oncogenic genes, promoting degradation of such transcripts [109].

In another mechanism, downregulated 5’ tsRNA-Leu (referred to as tRF-Leu-CCA-004) was found to modulate the expression of members of the cytochrome P450 family leading to the speculation that it could affect protein expression [110]. In the archaea *haloferax volcanii*, the 5’ tsRNA Val (referred to as tRF-5Val) binds to the small ribosomal subunit and interferes with the peptidyl transferase, hence destabilising the growing polypeptide chain [28]. On the contrary, a 3’ tRNA-Thr half in *Tripanosoma bruceii* was found to facilitate ribosomal-mRNA loading [111].

tsRNAs can also indirectly affect the level of protein translation by regulating RNA and protein ribosomal components. 3’ tsRNAs bearing m^1^A and pseudouridine marks can modulate rRNA expression via binding the Tetrahymena piWI 12 (Twi12) complex, Twi12-Xrn2-Tan1 [112]. Kim et al. identified a mechanism of ribosomal protein regulation driven by 3’ tsRNA-Leu^CAG^ [113]. 3’ tsRNA-Leu^CAG^ binds and unwinds the mRNA of ribosomal components, RPS28 and RPS15, promoting their translation and ultimately ribosomal biogenesis [113].

These studies suggest that tsRNAs can employ various mechanisms to drive inhibition of protein translation, offering yet another layer of gene expression regulation.

#### Regulation of the cell cycle and proliferation

Cancer is often mediated by dysregulation of the cell cycle via turning off checkpoints, leading to uncontrolled cell growth [114]. Apoptosis is a self-induced mechanism of cell death that aims to eliminate malignant or damaged cells [115]. tsRNAs have been implicated in the regulation of the cell cycle, proliferation, and apoptosis.

Aurora kinase A (AURKA) is a serine/threonine kinase essential to regulate mitosis, in particular chromosome segregation and cell division. It is regarded as a potent oncogene [116]. 5’ tsRNA-Leu^CAG^ (referred to as tRF-Leu^CAG)^ was found to be upregulated and promote cell cycle progression in non-small cell lung cancer (NSCLC). When this 5’ tsRNA was inhibited, AURKA was downregulated, whereas its upregulation caused AURKA hyperactivity and promotion of cell cycle progression through G1 phase [117]. It was proposed that this effect could potentially be mediated by 5’ tsRNA-Leu^CAG^ interaction with miRNA-137 and miRNA-32, known regulators of AURKA [117].

3’ tsRNA-Ser^TGA^ (referred to as tRF-1001) has been identified as a regulator of cell proliferation [32]. Depletion of tRF-1001 by antisense oligonucleotides caused a reduction in DNA synthesis and accumulation of cells in G2 [32]. 3’ tsRNA-CCA ends were identified to be bound to Twi12, which is a known cell cycle regulator [112, 118]. Members of the wingless-related integration site (Wnt) pathway, including frizzled 3 receptors (FZD3), have been shown to be often dysregulated in many malignant and aggressive cancers, causing self-renewal and driving metastasis [119]. 5’ tRNA half Val^CAC^ (also known as AS-tDR-001430), inhibits FZD3 and it was proposed to suppress the downstream Wnt/*β*-catenin signalling pathway. This results in the inhibition of cell proliferation and reduced cancer progression [120].

The Notch signalling pathway, which supports colorectal cancer progression, can be modulated by a similar mechanism. 3’ tsRNA (also referred to as miR1280), derived from both tRNA-Leu and pre-miRNA1280, binds directly to the Notch ligand, jagged 2 (JAG2). Binding occurs at the 3’ UTR and results in the inhibition of the Notch signalling pathway. This affects downstream Gata1/3 mediated transcriptional regulation of miR200b, a master regulator of differentiation and cancer progression [121].

Breast cancer anti-oestrogen resistance 3 (BCAR3) has been associated with ovarian cancer cell proliferation. Zhou and colleagues identified that 5’ tsRNA-Glu downregulates BCAR3 via binding to the BCAR3 mRNA 3’ UTR, suppressing ovarian cancer cell proliferation [122]. Furthermore, tRNAs, as well as tsRNAs, have been shown to bind to cytochrome c, therefore can influence cell fate by preventing apoptosis [123].

A Dicer-dependent 3’ tsRNA (referred to as tRF-3 CU1276), was shown to have regulatory miRNA-like properties. It was also reported to repress the expression of a set of genes including *RPA1*, a master regulator of DNA dynamics and DNA damage response [41]. Lastly, the expression of 3’ tsRNA CU1276 in Burkitt lymphoma-derived cell lines drove the arrest of cell proliferation [41].

In summary, various examples of specific tsRNA-driven mechanisms of cell cycle progression and proliferation regulation make them highly interesting therapeutic targets for cancer therapy.

#### Intercellular communication

Intercellular communication is essential for mediating tissue organisation and homoeostasis, as well as eliciting responses throughout the body [124]. Exosomes are extracellular vesicles (EVs) which can carry cargo, of which sncRNAs, including tsRNAs, form a substantial part, between cells [125]. The cargo of EVs is tissue and cell-type dependent. For example, tRNA/tsRNAs constitute 5% of the total sncRNAs content of the exosomes released by liver cancer cells, of which 90% are 5’ tsRNAs of few enriched tRNA isotypes and they are significantly upregulated compared to healthy tissue samples [126].

Gambaro et al. showed that synthetic tsRNAs (5’ tRNA half Gly) could be transferred from donor to recipient cells [127], hence promoting the idea that tsRNAs could play intercellular roles via EV delivery. One of such intercellular mechanisms, driven by tsRNAs, is the regulation of T-cell activation [128]. Immune response activation is not only crucial for protecting against pathogen invasion, but also for recognition and elimination of cancer cells at the onset of malignant tumorigenesis [129]. tsRNAs were specifically enriched sncRNAs in EVs secreted by activated T cells compared to corresponding cellular RNA content and were differentially expressed compared to EVs from resting T cells [128]. Chiou et al. reported a T-cell activation regulatory feedback loop. Activated T cells promote inhibition of new T-cell activation via packing and releasing specific tsRNAs into EVs. In this model, EV packaging of tsRNAs functions to sequester specific inhibitory tsRNAs by compartmentalisation. However, this could also potentially mediate intercellular communication [128]. Lastly, the parasite *Schistosoma mansoni* was shown to release extracellular tsRNAs and hypothesised to regulate gene expression in the host cells upon EV uptake [130].

YBX1 is a good candidate for sorting and loading of specific tsRNAs into EVs because YBX1 sorts loading of other sncRNAs, such as specific miRNA molecules [131] and has also affinity for specific sets of tsRNAs with CU-box motif [108, 109]. However, YBX1 was shown to be dispensable in loading synthetic overexpressed tRNA halves in MCF-7-derived EVs [127]. Therefore, the role of YBX1 in tsRNAs and tRNA halves loading in EVs in endogenous setup requires more investigation.

#### Oncogenic and tumour suppressor tsRNAs

tsRNAs involved in different cancer types have been comprehensively reviewed previously [50]. Here, we report selective and relevant examples of these tsRNAs, many of which we have previously mentioned in this review. We have classified these examples as proto-oncogenic or tumour suppressor tsRNAs, shown together with the specific mechanism by which they function, if known, in Table 1.

### tsRNA-based therapeutic applications

Following the identification of tsRNAs as a bona fide novel class of regulatory sncRNAs, there has been keen interest in the use of tsRNAs for a plethora of potential therapeutic applications. Since a study by Balatti et al. brought to light that cancers may have specific tsRNA signatures [21], an increasing number of studies have analysed tsRNA expression levels in cancer cell lines, tissues, and extracellular samples [126, 132–142]. Balatti et al. predominantly identified type II tsRNAs [21]. This may be due to the fact that pre-tRNA-derived fragments are less decorated by chemical modifications, therefore are easier to detect by sequencing [27]. However, at present, there is no specific type of tsRNAs whose expression is predominantly dysregulated in cancer. This is likely due to the heterogeneity of mechanisms which tsRNAs can mediate, as discussed above.

Originally thought to be very low in abundance, the use of NGS techniques that have improved the detection of RNA, has shown that in fact tsRNAs (together with rRNA-derived small RNAs) are among the most predominant sncRNA classes in many cell types and tissues [5]. In addition, tsRNAs can be detected in bodily fluids, such as saliva and plasma, where they may be protected inside EVs that increase their stability [138]. For example, tsRNAs with diagnostic and prognostic values were identified in non-invasive breast cancer liquid biopsies [143]. Hence, tsRNAs have great potential as useful diagnostic and prognostic biomarkers. In Table 2, we report examples of some validated tsRNA cancer biomarkers.

**Table 2:** Examples of tsRNA biomarkers in cancer.

tsRNA name	Class of tsRNA	Biological relevance	Reference
ts-46, ts-47	pre-tRNA 3’ tsRNA	Downregulated in lung cancer; affect cell growth and survival	[21]
tRF-23-Q99P9P9NDD	5’ tsRNA	High expression is associated with a low survival rate in patients with gastric cancer; proposed to target genes: *BCKDHB, DGKD* and *GNPDA2*	[152]
tRF-20-S998LO9D	5’ tsRNA	Correlates with tumour progression via an unknown mechanism	[153]
tsRNA-ValTAC-41	3’ tRNA half	Upregulated in patients with pancreatic adenocarcinoma and in exosomes of liver cancer patients	[126, 142]
tsRNA-26576	Not classified	Upregulated in breast cancer, proposed to promote cellular growth, and inhibit apoptosis	[154]
5’ tiRNA-Pro^TGG^	5’ tRNA half	Downregulated in colorectal adenocarcinoma patients and associated with poor patient survival; proposed to regulate several pathways (AMPK, MAPK and mTOR)	[155]
tsRNA-5001a	Not classified	Upregulated in lung adenocarcinoma tissue, promotes the proliferation of lung adenocarcinoma cells	[156]
AS-tDR-007872	5’ tRNA half	Downregulated in lung cancer patients and regulates apoptosis by downregulating BCL2L11	[157]

However, major limitations in the use of tsRNAs as biomarkers still exist. Firstly, despite the advances in NGS techniques that have allowed the detection of chemically modified RNAs, such as tsRNAs, there is still limited availability of such tools. Secondly, sequence similarity between tsRNAs and full-length precursors could mislead the detection of tsRNA sequences [27]. Lastly, more studies are required to confirm the accuracy of tsRNA expression levels as biomarkers for diagnostic and prognostic applications.

Oligonucleotide-based therapy utilises short oligonucleotides to modulate gene expression via RNAi mechanisms. It is an active area of drug design, targeting gene-specific pathologies [144]. siRNA-based therapies have been recently FDA-approved and implemented in the clinic [145]. Studies have shown several gene silencing mechanisms driven by tsRNAs, as discussed above. We and others have utilised synthetic tsRNA mimics in the laboratory setup and have observed changes in RNA levels of the target genes [42, 120, 127]. However, while siRNAs are short RNA duplexes, tsRNAs are single-stranded RNAs, therefore are potentially more prone to degradation. To make tsRNAs an effective silencing strategy for therapeutic usage, chemical modifications of nucleotides, to increase their stability, as well as sequence optimisation, would be required to obtain silencing effects of comparable efficiency to siRNA, or greater.

Antisense oligonucleotide (ASO) technology is another emerging area of oligonucleotide-based therapy. It is based on the idea that endogenous sncRNAs can be sequestered by Watson–Crick base pairing with synthetic short oligonucleotides, promoting the ablation of RNAi and, consequently, gene activation [144]. ASOs against tsRNAs have been successfully used in a laboratory setting in a few studies [113, 128], but tsRNA ASOs face challenges. For example, ASOs are highly prone to degradation. Using modified nucleic acids, such as locked nucleic acid (LNA), increases the stability of ASOs without affecting their pharmacokinetic characteristics [144, 146]. Moreover, given the sequence similarity between tsRNAs and tRNAs, ASOs could potentially interfere with tRNA stability by binding to the full-length tRNAs. miRNA sponges are an example of longer ASO constructs designed with multiple binding sites to sequester malignant endogenous miRNAs [147]. Since miRNA and tsRNA share many similarities in structure and function, it is not surprising that similar ASO strategies are being considered for tsRNAs [27]. These longer ASOs not only have the advantage of not interfering with full-length tRNAs, but they also have increased stability, resistance against RNase H degradation and can accommodate multiple tsRNA sequences in one molecule.

Lastly, Onconase (ONC) is a small RNase that has great cellular uptake, resistance against RNase Inhibitors and a propensity for targeting cancer cells [148]. Since the first clinical trial in 1993, ONC has been administered to cancer patients alone or in combination with other treatments [148]. ONC functions by exerting regulatory roles, mainly regarding cell cycle arrest and induction of apoptosis [148]. It is not clear exactly how ONC exerts its anti-tumour effects, but it is very likely to be mediated by its ribonucleolytic cleavage activity. ONC cleaves tRNAs and other ncRNAs, such as pre-miRNAs, with a specific sequence and structure preference, mimicking ANG and Dicer. Hence, ONC could be useful for generating tsRNAs [148]. More research on the potential applications of ONC is required. Nevertheless, given that ONC has been safely used in clinical settings for some years, it would be very convenient to implement its usage for tsRNA-based therapeutic strategies.

## Concluding remarks

tsRNA is an exciting and promising new field in RNA and cancer biology. The growing number of studies showing tsRNA-driven mechanisms makes it clear that tRNA fragments are not just degradation products, but functional and powerful sncRNAs. We believe that efforts should be channelled towards investigating crucial aspects of tsRNAs which, at present, are not well-characterised. Firstly, the mechanisms of tsRNA biogenesis require further study. Mechanistic insights into tRNA cleavage and its regulation are key aspects of this. The cellular localisation of tsRNA biogenesis also requires further investigation since Dicer nuclear localisation has been reported [149]. Secondly, we need further exploration into the roles of the chemical modifications of tRNA (for tsRNA biogenesis) and tsRNAs (for stability and signalling). Lastly, the interplay between tsRNAs and other classes of sncRNAs deserves attention as it could unravel a novel regulatory and signalling network, revealing yet another layer of cellular and gene regulation. We predict that, in the next decade, an increasing volume of information surrounding tsRNA biology will be brought to light.

## References

[1] Holley R, Apgar J, Everett G, Madison J, Marquisee M, Merrill S (1965). Structure of a ribonucleic acid. Science..

[2] Hoagland MB, Stephenson ML, Scott JF, Hecht LI, Zamecnik PC (1958). A soluble ribonucleic acid intermediate in protein synthesis. J Biol Chem.

[3] Borek E, Baliga BS, Gehrke CW, Kuo CW, Belman S, Troll W (1977). High turnover rate of transfer RNA in tumor tissue. Cancer Res.

[4] Speer J, Gehrke CW, Kuo KC, Waalkes TP, Borek E (1979). tRNA breakdown products as markers for cancer. Cancer..

[5] Shi J, Zhang Y, Tan D, Zhang X, Yan M, Zhang Y (2021). PANDORA-seq expands the repertoire of regulatory small RNAs by overcoming RNA modifications. Nat Cell Biol.

[6] Leger A, Amaral PP, Pandolfini L, Capitanchik C, Capraro F, Miano V (2021). RNA modifications detection by comparative Nanopore direct RNA sequencing. Nat Commun.

[7] Cole C, Sobala A, Lu C, Thatcher SR, Bowman A, Brown JW (2009). Filtering of deep sequencing data reveals the existence of abundant Dicer-dependent small RNAs derived from tRNAs. RNA..

[8] Kawaji H, Nakamura M, Takahashi Y, Sandelin A, Katayama S, Fukuda S (2008). Hidden layers of human small RNAs. BMC Genomics.

[9] Babiarz JE, Ruby JG, Wang Y, Bartel DP, Blelloch R (2008). Mouse ES cells express endogenous shRNAs, siRNAs, and other microprocessor-independent, Dicer-dependent small RNAs. Genes Dev.

[10] Soares AR, Fernandes N, Reverendo M, Araújo HR, Oliveira JL, Moura GM (2015). Conserved and highly expressed tRNA derived fragments in zebrafish. BMC Mol Biol.

[11] Zuo Y, Zhu L, Guo Z, Liu W, Zhang J, Zeng Z (2021). tsRBase: a comprehensive database for expression and function of tsRNAs in multiple species. Nucleic Acids Res.

[12] Wang JH, Chen WX, Mei SQ, Yang YD, Yang JH, Qu LH (2022). tsRFun: a comprehensive platform for decoding human tsRNA expression, functions and prognostic value by high-throughput small RNA-Seq and CLIP-Seq data. Nucleic Acids Res.

[13] Yao D, Sun X, Zhou L, Amanullah M, Pan X, Liu Y (2020). OncotRF: an online resource for exploration of tRNA-derived fragments in human cancers. RNA Biol.

[14] Pliatsika V, Loher P, Magee R, Telonis AG, Londin E, Shigematsu M (2018). MINTbase v2.0: a comprehensive database for tRNA-derived fragments that includes nuclear and mitochondrial fragments from all The Cancer Genome Atlas projects. Nucleic Acids Res.

[15] Chan PP, Lowe TM (2016). GtRNAdb 2.0: an expanded database of transfer RNA genes identified in complete and draft genomes. Nucleic Acids Res.

[16] Dieci G, Conti A, Pagano A, Carnevali D (2013). Identification of RNA polymerase III-transcribed genes in eukaryotic genomes. Biochimica et Biophysica Acta (BBA)-Gene Regul Mech.

[17] Torres AG (2019). Enjoy the silence: nearly half of human tRNA genes are silent. Bioinforma Biol Insights.

[18] Ehrlich R, Davyt M, López I, Chalar C, Marín M (2021). On the track of the missing tRNA genes: a source of non-canonical functions?. Front Mol Biosci.

[19] Orellana EA, Siegal E, Gregory RI. tRNA dysregulation and disease. Nat Rev Genet. 2022;23:651–64. 10.1038/s41576-022-00501-9 PMC11170316 35681060

[20] Torres AG, Reina O, Stephan-Otto Attolini C, Ribas de Pouplana L (2019). Differential expression of human tRNA genes drives the abundance of tRNA-derived fragments. Proc Natl Acad Sci USA.

[21] Balatti V, Nigita G, Veneziano D, Drusco A, Stein GS, Messier TL (2017). tsRNA signatures in cancer. Proc Natl Acad Sci USA.

[22] Lee YS, Dutta A (2009). MicroRNAs in cancer. Annu Rev Pathol.

[23] Liu Y, Dou M, Song X, Dong Y, Liu S, Liu H (2019). The emerging role of the piRNA/piwi complex in cancer. Mol Cancer.

[24] Kessler AC, Maraia RJ (2021). The nuclear and cytoplasmic activities of RNA polymerase III, and an evolving transcriptome for surveillance. Nucleic Acids Res.

[25] Hopper AK, Phizicky EM (2003). tRNA transfers to the limelight. Genes Dev.

[26] Kumar P, Anaya J, Mudunuri SB, Dutta A (2014). Meta-analysis of tRNA derived RNA fragments reveals that they are evolutionarily conserved and associate with AGO proteins to recognize specific RNA targets. BMC Biol.

[27] Kim HK, Yeom JH, Kay MA (2020). Transfer RNA-derived small RNAs: another layer of gene regulation and novel targets for disease therapeutics. Mol Ther.

[28] Gebetsberger J, Zywicki M, Künzi A, Polacek N (2012). tRNA-derived fragments target the ribosome and function as regulatory non-coding RNA in *Haloferax volcanii*. Archaea..

[29] Zhang X, Cozen AE, Liu Y, Chen Q, Lowe TM (2016). Small RNA modifications: integral to function and disease. Trends Mol Med.

[30] Chen Q, Zhang X, Shi J, Yan M, Zhou T (2021). Origins and evolving functionalities of tRNA-derived small RNAs. Trends Biochem Sci.

[31] Haussecker D, Huang Y, Lau A, Parameswaran P, Fire AZ, Kay MA (2010). Human tRNA-derived small RNAs in the global regulation of RNA silencing. RNA..

[32] Lee YS, Shibata Y, Malhotra A, Dutta A (2009). A novel class of small RNAs: tRNA-derived RNA fragments (tRFs). Genes Dev.

[33] Telonis AG, Loher P, Honda S, Jing Y, Palazzo J, Kirino Y (2015). Dissecting tRNA-derived fragment complexities using personalized transcriptomes reveals novel fragment classes and unexpected dependencies. Oncotarget..

[34] Hanada T, Weitzer S, Mair B, Bernreuther C, Wainger BJ, Ichida J (2013). CLP1 links tRNA metabolism to progressive motor-neuron loss. Nature..

[35] Su Z, Wilson B, Kumar P, Dutta A (2020). Noncanonical roles of tRNAs: tRNA fragments and beyond. Annu Rev Genet.

[36] Soares AR, Santos M. Discovery and function of transfer RNA-derived fragments and their role in disease. Wiley Interdiscip Rev RNA. 2017;8:e1423. 10.1002/wrna.1423 28608481

[37] Yamasaki S, Ivanov P, Hu GF, Anderson P (2009). Angiogenin cleaves tRNA and promotes stress-induced translational repression. J Cell Biol.

[38] Kikuchi Y, Sasaki N, Ando-Yamagami Y (1990). Cleavage of tRNA within the mature tRNA sequence by the catalytic RNA of RNase P: implication for the formation of the primer tRNA fragment for reverse transcription in copia retrovirus-like particles. Proc Natl Acad Sci USA.

[39] Fu H, Feng J, Liu Q, Sun F, Tie Y, Zhu J (2009). Stress induces tRNA cleavage by angiogenin in mammalian cells. FEBS Lett.

[40] Su Z, Kuscu C, Malik A, Shibata E, Dutta A (2019). Angiogenin generates specific stress-induced tRNA halves and is not involved in tRF-3-mediated gene silencing. J Biol Chem.

[41] Maute RL, Schneider C, Sumazin P, Holmes A, Califano A, Basso K (2013). tRNA-derived microRNA modulates proliferation and the DNA damage response and is down-regulated in B cell lymphoma. Proc Natl Acad Sci USA.

[42] Di Fazio A, Schlackow M, Pong SK, Alagia A, Gullerova M (2022). Dicer dependent tRNA derived small RNAs promote nascent RNA silencing. Nucleic Acids Res.

[43] Megel C, Hummel G, Lalande S, Ubrig E, Cognat V, Morelle G (2019). Plant RNases T2, but not Dicer-like proteins, are major players of tRNA-derived fragments biogenesis. Nucleic Acids Res.

[44] Schaefer M, Pollex T, Hanna K, Tuorto F, Meusburger M, Helm M (2010). RNA methylation by Dnmt2 protects transfer RNAs against stress-induced cleavage. Genes Dev.

[45] Liu S, Chen Y, Ren Y, Zhou J, Ren J, Lee I (2018). A tRNA-derived RNA Fragment Plays an Important Role in the Mechanism of Arsenite -induced Cellular Responses. Sci Rep..

[46] Langenberger D, Cakir MV, Hoffmann S, Stadler PF (2013). Dicer‐processed small RNAs: rules and exceptions. J Exp Zool Part B: Mol Dev Evol.

[47] Kuscu C, Kumar P, Kiran M, Su Z, Malik A, Dutta A (2018). tRNA fragments (tRFs) guide Ago to regulate gene expression post-transcriptionally in a Dicer-independent manner. Rna..

[48] Li Z, Ender C, Meister G, Moore PS, Chang Y, John B (2012). Extensive terminal and asymmetric processing of small RNAs from rRNAs, snoRNAs, snRNAs, and tRNAs. Nucleic Acids Res.

[49] Jia Y, Tan W, Zhou Y (2020). Transfer RNA-derived small RNAs: potential applications as novel biomarkers for disease diagnosis and prognosis. Ann Transl Med.

[50] Wang Y, Weng Q, Ge J, Zhang X, Guo J, Ye G (2022). tRNA-derived small RNAs: mechanisms and potential roles in cancers. Genes Dis..

[51] La Ferlita A, Alaimo S, Veneziano D, Nigita G, Balatti V, Croce CM (2019). Identification of tRNA-derived ncRNAs in TCGA and NCI-60 panel cell lines and development of the public database tRFexplorer. Database.

[52] Dhahbi JM, Spindler SR, Atamna H, Boffelli D, Martin DI (2014). Deep sequencing of serum small RNAs identifies patterns of 5’ tRNA half and YRNA fragment expression associated with breast cancer. Biomark Cancer.

[53] Zheng LL, Xu WL, Liu S, Sun WJ, Li JH, Wu J (2016). tRF2Cancer: a web server to detect tRNA-derived small RNA fragments (tRFs) and their expression in multiple cancers. Nucleic Acids Res.

[54] Grewal SS (2015). Why should cancer biologists care about tRNAs? tRNA synthesis, mRNA translation and the control of growth. Biochim Biophys Acta.

[55] Zhang X, He X, Liu C, Liu J, Hu Q, Pan T (2016). IL-4 inhibits the biogenesis of an epigenetically suppressive PIWI-interacting RNA to upregulate CD1a molecules on monocytes/dendritic cells. J Immunol.

[56] Liu Z, Kim HK, Xu J, Jing Y, Kay MA (2021). The 3’tsRNAs are aminoacylated: implications for their biogenesis. PLoS Genet.

[57] Lin TC (2022). RUNX1 and cancer. Biochim Biophys Acta Rev Cancer.

[58] Farina NH, Scalia S, Adams CE, Hong D, Fritz AJ, Messier TL (2020). Identification of tRNA-derived small RNA (tsRNA) responsive to the tumor suppressor, RUNX1, in breast cancer. J Cell Physiol.

[59] Ontiveros RJ, Stoute J, Liu KF (2019). The chemical diversity of RNA modifications. Biochem J.

[60] Lorenz C, Lünse CE, Mörl M (2017). tRNA modifications: impact on structure and thermal adaptation. Biomolecules..

[61] Väre VY, Eruysal ER, Narendran A, Sarachan KL, Agris PF (2017). Chemical and conformational diversity of modified nucleosides affects tRNA structure and function. Biomolecules..

[62] Boccaletto P, Stefaniak F, Ray A, Cappannini A, Mukherjee S, Purta E (2022). MODOMICS: a database of RNA modification pathways. 2021 update. Nucleic Acids Res.

[63] Pan T (2018). Modifications and functional genomics of human transfer RNA. Cell Res.

[64] de Crécy-Lagard V, Boccaletto P, Mangleburg CG, Sharma P, Lowe TM, Leidel SA (2019). Matching tRNA modifications in humans to their known and predicted enzymes. Nucleic Acids Res.

[65] Wang X, Matuszek Z, Huang Y, Parisien M, Dai Q, Clark W (2018). Queuosine modification protects cognate tRNAs against ribonuclease cleavage. Rna..

[66] Blanco S, Bandiera R, Popis M, Hussain S, Lombard P, Aleksic J (2016). Stem cell function and stress response are controlled by protein synthesis. Nature..

[67] Blanco S, Dietmann S, Flores JV, Hussain S, Kutter C, Humphreys P (2014). Aberrant methylation of tRNAs links cellular stress to neuro-developmental disorders. EMBO J.

[68] Guzzi N, Cieśla M, Ngoc PCT, Lang S, Arora S, Dimitriou M (2018). Pseudouridylation of tRNA-derived fragments steers translational control in stem cells. Cell..

[69] Bohnsack KE, Höbartner C, Bohnsack MT (2019). Eukaryotic 5-methylcytosine (m5C) RNA methyltransferases: mechanisms, cellular functions, and links to disease. Genes..

[70] Tuorto F, Liebers R, Musch T, Schaefer M, Hofmann S, Kellner S (2012). RNA cytosine methylation by Dnmt2 and NSun2 promotes tRNA stability and protein synthesis. Nat Struct Mol Biol.

[71] Yu T, Xie Y, Tang C, Wang Y, Yuan S, Zheng H (2021). Dnmt2-null sperm block maternal transmission of a paramutant phenotype†. Biol Reprod.

[72] Zhang Y, Zhang X, Shi J, Tuorto F, Li X, Liu Y (2018). Dnmt2 mediates intergenerational transmission of paternally acquired metabolic disorders through sperm small non-coding RNAs. Nat Cell Biol.

[73] Durdevic Z, Mobin MB, Hanna K, Lyko F, Schaefer M (2013). The RNA methyltransferase Dnmt2 is required for efficient Dicer-2-dependent siRNA pathway activity in Drosophila. Cell Rep..

[74] Wang L, Zhang J, Su Y, Maimaitiyiming Y, Yang S, Shen Z (2022). Distinct roles of m(5)C RNA methyltransferase NSUN2 in major gynecologic cancers. Front Oncol.

[75] Okamoto M, Fujiwara M, Hori M, Okada K, Yazama F, Konishi H (2014). tRNA modifying enzymes, NSUN2 and METTL1, determine sensitivity to 5-fluorouracil in HeLa cells. PLoS Genet.

[76] Yi J, Gao R, Chen Y, Yang Z, Han P, Zhang H (2017). Overexpression of NSUN2 by DNA hypomethylation is associated with metastatic progression in human breast cancer. Oncotarget..

[77] Xiang S, Ma Y, Shen J, Zhao Y, Wu X, Li M (2020). m(5)C RNA methylation primarily affects the ErbB and PI3K-Akt signaling pathways in gastrointestinal cancer. Front Mol Biosci.

[78] Su J, Wu G, Ye Y, Zhang J, Zeng L, Huang X (2021). NSUN2-mediated RNA 5-methylcytosine promotes esophageal squamous cell carcinoma progression via LIN28B-dependent GRB2 mRNA stabilization. Oncogene..

[79] Frye M, Dragoni I, Chin SF, Spiteri I, Kurowski A, Provenzano E (2010). Genomic gain of 5p15 leads to over-expression of Misu (NSUN2) in breast cancer. Cancer Lett.

[80] Hussain S, Sajini AA, Blanco S, Dietmann S, Lombard P, Sugimoto Y (2013). NSun2-mediated cytosine-5 methylation of vault noncoding RNA determines its processing into regulatory small RNAs. Cell Rep..

[81] Sajini AA, Choudhury NR, Wagner RE, Bornelöv S, Selmi T, Spanos C (2019). Loss of 5-methylcytosine alters the biogenesis of vault-derived small RNAs to coordinate epidermal differentiation. Nat Commun.

[82] Pereira M, Ribeiro DR, Pinheiro MM, Ferreira M, Kellner S, Soares AR (2021). m5U54 tRNA hypomodification by lack of TRMT2A drives the generation of tRNA-derived small RNAs. Int J Mol Sci.

[83] He C, Bozler J, Janssen KA, Wilusz JE, Garcia BA, Schorn AJ (2021). TET2 chemically modifies tRNAs and regulates tRNA fragment levels. Nat Struct Mol Biol.

[84] Chen Z, Qi M, Shen B, Luo G, Wu Y, Li J (2019). Transfer RNA demethylase ALKBH3 promotes cancer progression via induction of tRNA-derived small RNAs. Nucleic Acids Res.

[85] Song J, Zhuang Y, Zhu C, Meng H, Lu B, Xie B (2020). Differential roles of human PUS10 in miRNA processing and tRNA pseudouridylation. Nat Chem Biol.

[86] Cui Q, Yin K, Zhang X, Ye P, Chen X, Chao J (2021). Targeting PUS7 suppresses tRNA pseudouridylation and glioblastoma tumorigenesis. Nat Cancer.

[87] Guzzi N, Muthukumar S, Cieśla M, Todisco G, Ngoc PCT, Madej M (2022). Pseudouridine-modified tRNA fragments repress aberrant protein synthesis and predict leukaemic progression in myelodysplastic syndrome. Nat Cell Biol.

[88] Holcik M, Sonenberg N (2005). Translational control in stress and apoptosis. Nat Rev Mol Cell Biol.

[89] Cristodero M, Brogli R, Joss O, Schimanski B, Schneider A, Polacek N (2021). tRNA 3’ shortening by LCCR4 as a response to stress in *Trypanosoma brucei*. Nucleic Acids Res.

[90] Ivanov P, Emara MM, Villen J, Gygi SP, Anderson P (2011). Angiogenin-induced tRNA fragments inhibit translation initiation. Mol Cell.

[91] Thompson DM, Parker R (2009). Stressing out over tRNA cleavage. Cell..

[92] Honda S, Loher P, Shigematsu M, Palazzo JP, Suzuki R, Imoto I (2015). Sex hormone-dependent tRNA halves enhance cell proliferation in breast and prostate cancers. Proc Natl Acad Sci USA.

[93] Liu J, Carmell MA, Rivas FV, Marsden CG, Thomson JM, Song JJ (2004). Argonaute2 is the catalytic engine of mammalian RNAi. Science..

[94] Jonas S, Izaurralde E (2015). Towards a molecular understanding of microRNA-mediated gene silencing. Nat Rev Genet.

[95] Castel SE, Martienssen RA (2013). RNA interference in the nucleus: roles for small RNAs in transcription, epigenetics and beyond. Nat Rev Genet.

[96] Martienssen R, Moazed D (2015). RNAi and heterochromatin assembly. Cold Spring Harb Perspect Biol.

[97] Wang H, Huang W, Fan X, He X, Chen S, Yu S (2022). The tRNA-derived fragment tRF-24-V29K9UV3IU functions as a miRNA-like RNA to prevent gastric cancer progression by inhibiting GPR78 expression. J Oncol.

[98] Schopman NC, Heynen S, Haasnoot J, Berkhout B (2010). A miRNA-tRNA mix-up: tRNA origin of proposed miRNA. RNA Biol.

[99] Park J, Ahn SH, Shin MG, Kim HK, Chang S (2020). tRNA-derived small RNAs: novel epigenetic regulators. Cancers.

[100] Hussain S, Tulsyan S, Dar SA, Sisodiya S, Abiha U, Kumar R (2022). Role of epigenetics in carcinogenesis: recent advancements in anticancer therapy. Semin Cancer Biol.

[101] Pekarsky Y, Balatti V, Palamarchuk A, Rizzotto L, Veneziano D, Nigita G (2016). Dysregulation of a family of short noncoding RNAs, tsRNAs, in human cancer. Proc Natl Acad Sci USA.

[102] Burns KH (2017). Transposable elements in cancer. Nat Rev Cancer.

[103] Schorn AJ, Gutbrod MJ, LeBlanc C, Martienssen R (2017). LTR-retrotransposon control by tRNA-derived small RNAs. Cell..

[104] Dai Z, Ramesh V, Locasale JW (2020). The evolving metabolic landscape of chromatin biology and epigenetics. Nat Rev Genet.

[105] Boskovic A, Bing XY, Kaymak E, Rando OJ (2020). Control of noncoding RNA production and histone levels by a 5’ tRNA fragment. Genes Dev.

[106] Boroughs LK, DeBerardinis RJ (2015). Metabolic pathways promoting cancer cell survival and growth. Nat Cell Biol.

[107] Sobala A, Hutvagner G (2013). Small RNAs derived from the 5’ end of tRNA can inhibit protein translation in human cells. RNA Biol.

[108] Lyons SM, Achorn C, Kedersha NL, Anderson PJ, Ivanov P (2016). YB-1 regulates tiRNA-induced stress granule formation but not translational repression. Nucleic Acids Res.

[109] Goodarzi H, Liu X, Nguyen HC, Zhang S, Fish L, Tavazoie SF (2015). Endogenous tRNA-derived fragments suppress breast cancer progression via YBX1 displacement. Cell..

[110] Lu H, Liu L, Han S, Wang B, Qin J, Bu K (2021). Expression of tiRNA and tRF in APP/PS1 transgenic mice and the change of related proteins expression. Ann Transl Med.

[111] Fricker R, Brogli R, Luidalepp H, Wyss L, Fasnacht M, Joss O (2019). A tRNA half modulates translation as stress response in *Trypanosoma brucei*. Nat Commun.

[112] Couvillion MT, Bounova G, Purdom E, Speed TP, Collins K (2012). A Tetrahymena Piwi bound to mature tRNA 3’ fragments activates the exonuclease Xrn2 for RNA processing in the nucleus. Mol Cell.

[113] Kim HK, Fuchs G, Wang S, Wei W, Zhang Y, Park H (2017). A transfer-RNA-derived small RNA regulates ribosome biogenesis. Nature..

[114] Stewart ZA, Westfall MD, Pietenpol JA (2003). Cell-cycle dysregulation and anticancer therapy. Trends Pharm Sci.

[115] Lowe SW, Lin AW (2000). Apoptosis in cancer. Carcinogenesis..

[116] Tang A, Gao K, Chu L, Zhang R, Yang J, Zheng J (2017). Aurora kinases: novel therapy targets in cancers. Oncotarget..

[117] Shao Y, Sun Q, Liu X, Wang P, Wu R, Ma Z (2017). tRF-Leu-CAG promotes cell proliferation and cell cycle in non-small cell lung cancer. Chem Biol Drug Des.

[118] Couvillion MT, Lee SR, Hogstad B, Malone CD, Tonkin LA, Sachidanandam R (2009). Sequence, biogenesis, and function of diverse small RNA classes bound to the Piwi family proteins of *Tetrahymena thermophila*. Genes Dev.

[119] Li C, Nguyen V, Clark KN, Zahed T, Sharkas S, Filipp FV (2019). Down-regulation of FZD3 receptor suppresses growth and metastasis of human melanoma independently of canonical WNT signaling. Proc Natl Acad Sci USA.

[120] Mo D, Jiang P, Yang Y, Mao X, Tan X, Tang X (2019). A tRNA fragment, 5’-tiRNA(Val), suppresses the Wnt/β-catenin signaling pathway by targeting FZD3 in breast cancer. Cancer Lett.

[121] Huang B, Yang H, Cheng X, Wang D, Fu S, Shen W (2017). tRF/miR-1280 suppresses stem cell-like cells and metastasis in colorectal cancer. Cancer Res.

[122] Zhou K, Diebel KW, Holy J, Skildum A, Odean E, Hicks DA (2017). A tRNA fragment, tRF5-Glu, regulates BCAR3 expression and proliferation in ovarian cancer cells. Oncotarget..

[123] Saikia M, Jobava R, Parisien M, Putnam A, Krokowski D, Gao XH (2014). Angiogenin-cleaved tRNA halves interact with cytochrome c, protecting cells from apoptosis during osmotic stress. Mol Cell Biol.

[124] Berumen Sánchez G, Bunn KE, Pua HH, Rafat M (2021). Extracellular vesicles: mediators of intercellular communication in tissue injury and disease. Cell Commun Signal.

[125] Tosar JP, Segovia M, Castellano M, Gámbaro F, Akiyama Y, Fagúndez P (2020). Fragmentation of extracellular ribosomes and tRNAs shapes the extracellular RNAome. Nucleic Acids Res.

[126] Zhu L, Li J, Gong Y, Wu Q, Tan S, Sun D (2019). Exosomal tRNA-derived small RNA as a promising biomarker for cancer diagnosis. Mol Cancer.

[127] Gámbaro F, Li Calzi M, Fagúndez P, Costa B, Greif G, Mallick E (2020). Stable tRNA halves can be sorted into extracellular vesicles and delivered to recipient cells in a concentration-dependent manner. RNA Biol.

[128] Chiou NT, Kageyama R, Ansel KM (2018). Selective export into extracellular vesicles and function of tRNA fragments during T cell activation. Cell Rep..

[129] Gonzalez H, Hagerling C, Werb Z (2018). Roles of the immune system in cancer: from tumor initiation to metastatic progression. Genes Dev.

[130] Nowacki FC, Swain MT, Klychnikov OI, Niazi U, Ivens A, Quintana JF (2015). Protein and small non-coding RNA-enriched extracellular vesicles are released by the pathogenic blood fluke Schistosoma mansoni. J Extracell Vesicles.

[131] Liu XM, Ma L, Schekman R (2021). Selective sorting of microRNAs into exosomes by phase-separated YBX1 condensates. eLife..

[132] Jin F, Yang L, Wang W, Yuan N, Zhan S, Yang P (2021). A novel class of tsRNA signatures as biomarkers for diagnosis and prognosis of pancreatic cancer. Mol Cancer.

[133] Chen H, Xu Z, Cai H, Peng Y, Yang L, Wang Z (2022). Identifying differentially expressed tRNA-derived small fragments as a biomarker for the progression and metastasis of colorectal cancer. Dis Markers.

[134] Fu BF, Xu CY (2022). Transfer RNA-derived small RNAs: novel regulators and biomarkers of cancers. Front Oncol.

[135] Gao L, Xu L, Wang X (2022). A systematic review of tRNA-derived small non-coding RNAs as diagnostic and prognostic markers in cancer. Technol Cancer Res Treat.

[136] Huang LT, Cui M, Silva M, Okuda K, Shimada Y, Wang JH (2022). Expression profiles of tRNA-derived fragments and their potential roles in lung adenocarcinoma. Ann Transl Med.

[137] Kishore C, Karunagaran D (2022). Non-coding RNAs as emerging regulators and biomarkers in colorectal cancer. Mol Cell Biochem.

[138] Li K, Lin Y, Luo Y, Xiong X, Wang L, Durante K (2022). A signature of saliva-derived exosomal small RNAs as predicting biomarker for esophageal carcinoma: a multicenter prospective study. Mol Cancer.

[139] Lu Z, Su K, Wang X, Zhang M, Ma S, Li H (2021). Expression profiles of tRNA-derived small RNAs and their potential roles in primary nasopharyngeal carcinoma. Front Mol Biosci.

[140] Shen Y, Yu X, Ruan Y, Li Z, Xie Y, Yan Z (2021). Global profile of tRNA-derived small RNAs in gastric cancer patient plasma and identification of tRF-33-P4R8YP9LON4VDP as a new tumor suppressor. Int J Med Sci..

[141] Sun X, Yang J, Yu M, Yao D, Zhou L, Li X (2020). Global identification and characterization of tRNA-derived RNA fragment landscapes across human cancers. NAR Cancer..

[142] Xue M, Shi M, Xie J, Zhang J, Jiang L, Deng X (2021). Serum tRNA-derived small RNAs as potential novel diagnostic biomarkers for pancreatic ductal adenocarcinoma. Am J Cancer Res.

[143] Wang J, Ma G, Ge H, Han X, Mao X, Wang X (2021). Circulating tRNA-derived small RNAs (tsRNAs) signature for the diagnosis and prognosis of breast cancer. NPJ Breast Cancer.

[144] Chi X, Gatti P, Papoian T (2017). Safety of antisense oligonucleotide and siRNA-based therapeutics. Drug Discov Today.

[145] Adams D, Gonzalez-Duarte A, O’Riordan WD, Yang CC, Ueda M, Kristen AV (2018). Patisiran, an RNAi therapeutic, for hereditary transthyretin amyloidosis. N. Engl J Med.

[146] Crooke ST, Baker BF, Crooke RM, Liang XH (2021). Antisense technology: an overview and prospectus. Nat Rev Drug Discov.

[147] Diener C, Keller A, Meese E (2022). Emerging concepts of miRNA therapeutics: from cells to clinic. Trends Genet.

[148] Menegazzi M, Gotte G (2022). Role of the ribonuclease ONCONASE in miRNA biogenesis and tRNA processing: focus on cancer and viral infections. Int J Mol Sci.

[149] Burger K, Gullerova M (2018). Nuclear re-localization of Dicer in primary mouse embryonic fibroblast nuclei following DNA damage. PLoS Genet.

[150] Tong L, Zhang W, Qu B, Zhang F, Wu Z, Shi J (2020). The tRNA-derived fragment-3017A promotes metastasis by inhibiting NELL2 in human gastric cancer. Front Oncol.

[151] Xu W, Zhou B, Wang J, Tang L, Hu Q, Wang J (2021). tRNA-derived fragment tRF-Glu-TTC-027 regulates the progression of gastric carcinoma via MAPK signaling pathway. Front Oncol.

[152] Zhang Y, Gu X, Qin X, Huang Y, Ju S (2022). Evaluation of serum tRF-23-Q99P9P9NDD as a potential biomarker for the clinical diagnosis of gastric cancer. Mol Med.

[153] Ma J, Liu F (2022). Study of tRNA-derived fragment tRF-20-S998LO9D in pan-cancer. Dis Markers.

[154] Zhou J, Wan F, Wang Y, Long J, Zhu X (2019). Small RNA sequencing reveals a novel tsRNA-26576 mediating tumorigenesis of breast cancer. Cancer Manag Res.

[155] Tsiakanikas P, Adamopoulos PG, Tsirba D, Artemaki PI, Papadopoulos IN, Kontos CK (2022). High expression of a tRNA(Pro) derivative associates with poor survival and independently predicts colorectal cancer recurrence. Biomedicines..

[156] Hu F, Niu Y, Mao X, Cui J, Wu X, Simone CB (2021). tsRNA-5001a promotes proliferation of lung adenocarcinoma cells and is associated with postoperative recurrence in lung adenocarcinoma patients. Transl Lung Cancer Res.

[157] Fan H, Liu H, Lv Y, Song Y (2022). AS-tDR-007872: a novel tRNA-derived small RNA acts an important role in non-small-cell lung cancer. Comput Math Methods Med.

